# Central nervous system miliary metastasis in breast cancer: a case series analysis and proposed identification criteria of a rare metastasis subtype

**DOI:** 10.1038/s41416-020-1008-2

**Published:** 2020-08-04

**Authors:** Sami I. Bashour, Nuhad K. Ibrahim, Donald F. Schomer, Kenneth R. Hess, Chao Gao, Debu Tripathy, Gregory N. Fuller

**Affiliations:** 1 Department of Breast Medical Oncology, The University of Texas MD Anderson Cancer Center, Houston, TX USA; 2 Department of Neuroradiology, The University of Texas MD Anderson Cancer Center, Houston, TX USA; 3 Department of Biostatistics, The University of Texas MD Anderson Cancer Center, Houston, TX USA; 4 Department of Radiation Oncology, The Fourth Hospital of Hebei Medical University, Shijiazhuang, China; 5 Department of Anatomical Pathology, Section of Neuropathology, The University of Texas MD Anderson Cancer Center, Houston, TX USA

**Keywords:** Breast cancer, Cancer imaging, Metastasis, CNS cancer

## Abstract

**Background:**

CNS miliary metastasis (MiM) is poorly recognised in breast and other malignancies. Given its rarity, little epidemiologic, radiographic and clinical data are known. Although usually identified on neuroimaging, criteria for radiographic diagnosis do not exist. In this analysis, we establish its presence in breast cancer and identify factors contributing to outcome.

**Methods:**

We identified 546 female patients with brain metastasis from breast cancer between 2000 and 2015. Radiographic criteria were established through review of neuroimages by a senior Neuroradiologist, and defined as: (1) ≥20 lesions per image on ≥2 non-contiguous MRI images or ≥10 lesions per image on ≥2 non-contiguous CT images, and (2) bilateral lesions located in both the supratentorial and infratentorial compartments.

**Results:**

Twenty-one MiM cases were identified (3.8%). Number and anatomical distribution of metastases best identified MiM, while lesion size did not. Ten patients were diagnosed with MiM as initial CNS metastasis; 11 developed MiM following known CNS metastasis. Breast cancer subtype did not influence MiM development before or after other CNS metastasis.

**Conclusions:**

This is the first study to propose radiographic criteria for MiM diagnosis. Additional analysis is needed to verify data, but our results may enable a standardised approach for future MiM research.

## Background

Central nervous system (CNS) metastases are documented in up to 20% of breast cancer patients with advanced disease.^1^ Rates are increasing, however, as technological advancements increase imaging modality accuracy and, in tandem, newer systemic treatment regimens create sanctuary sites within the CNS.^2^ Historically, the most commonly described subset of CNS metastatic disease comprises lesions located at the grey-white matter junction that grow as expanding spherical masses; the anatomic location has been ascribed to hematogenous seeding in which tumour emboli become lodged at the point where blood vessel diameter approaches that of the metastatic cancer cell.^3^ Rarely documented and poorly understood, however, is a pathophysiologically different subset of CNS metastatic disease referred to as miliary metastasis (MiM).

First described as “carcinomatous encephalitis” by Madow and Alpers in 1951^4^ due to the nonspecific neurologic signs and symptoms at presentation that mimicked a toxic-metabolic encephalopathic state, MiM has largely been reported in the context of primary lung and gastrointestinal adenocarcinoma,^5,6^ with occasional instances of melanoma,^7^ and rare associations with breast cancer.^8,9^ Moreover, although most frequently identified by neuroimaging, individual case reports of MiM have thus far been published only with general descriptions of CNS radiographic findings following autopsy and histopathologic review. To date, radiographic criteria to objectively diagnose MiM do not exist, and published case reports typically only note numerous foci with a perivascular microscopic anatomic distribution, a propensity for initiation within the cortical grey ribbon rather than at the grey-white junction, and a relative paucity of typical intraparenchymal lesions.^10–12^ Given the rarity of MiM, however, few radiographic and clinical studies have thus far been conducted with the aim of elucidating disease outcomes or prognostic factors.

The goal of this single-institution analysis of patients with brain metastasis from primary breast cancer is to better understand MiM through the identification of clinical factors that contribute to disease outcomes and prognosis.

## Methods

This single-institution study was approved by the Institutional Review Board of The University of Texas MD Anderson Cancer Center, a high-volume tertiary cancer centre. From the institution’s prospectively maintained electronic database, we reviewed records and images of female patients diagnosed or evaluable with brain metastasis from primary breast cancer between 2000 and 2015; patients diagnosed with brain metastases prior to 2000 and no documented CNS recurrence after 2000 were excluded due to the inaccessibility of neuroimaging studies in the electronic medical record (only the radiographic reports are available for review in cases prior to 2000). Patients were considered eligible for inclusion only if neuroimaging by computerised tomography (CT) or magnetic resonance imaging (MRI) was available for direct review by a neuroradiologist. Both CT and MRI studies included post-contrast imaging; iodinated contrast was utilised for CT imaging and gadolinium chelate was used for MR imaging. Neuroimages of all patients were reviewed by the same Neuroradiologist (DFS). Patients with leptomeningeal disease (LMD) or skull-based only metastasis but no documented intraparenchymal lesions on imaging were excluded from this review. Patients with CNS disease recurrence only at the site of prior surgical intervention were also excluded. Tissue biopsy and histopathologic confirmation of brain metastases were not available in all patients, but brain metastases were attributed to breast cancer in the clinical context of active disease progression on imaging, including in the brain. In instances where patients were noted to have a history of multiple primary cancers, brain metastases were attributed to breast cancer only if tissue confirmation was available.

Upon direct review of all neuroimaging by the study Neuroradiologist (DFS), we established two principal radiographic criteria for MiM diagnosis: (1) at least 20 metastatic lesions per image slice on 2 or more non-contiguous image slices by MRI, or at least 10 lesions per image slice on 2 or more non-contiguous image slices by CT, and (2) MiM lesions are required to be present bilaterally and in both the supratentorial and infratentorial compartments. The anatomical distribution and number of lesions were recorded, and patients were stratified into groups based on the presence or absence of traditional intraparenchymal metastases, dural-based metastases, and/or MiM.

Patients were evaluated according to breast cancer subtype and divided into four groups: those with hormone receptor–positive and human epithelial growth factor 2–positive tumours (HR+/HER2+), HR-positive but HER2-negative tumours (HR+/HER2−), HR-negative but HER2-positive tumours (HR−/HER2+), and HR-negative and HER2-negative tumours (HR−/HER2−), more commonly known as triple-negative breast cancer. HR+ status was defined as either oestrogen or progesterone receptor positivity by institutional criteria; institutional criteria was also used for HER2 status. Time to CNS metastasis, defined as time from primary breast cancer diagnosis to first known CNS involvement, and time to MiM diagnosis, defined as time from breast cancer diagnosis to development of MiM based on radiographic criteria, were determined. Median time to MiM development and overall survival after MiM diagnosis were analysed according to breast cancer subtype. Log rank tests were used for statistical analysis between breast cancer subtype groups, and to determine statistically significant differences. A Kaplan–Meier curve was used to represent overall survival according to breast cancer subtype.

## Results

**Fig. 1:**
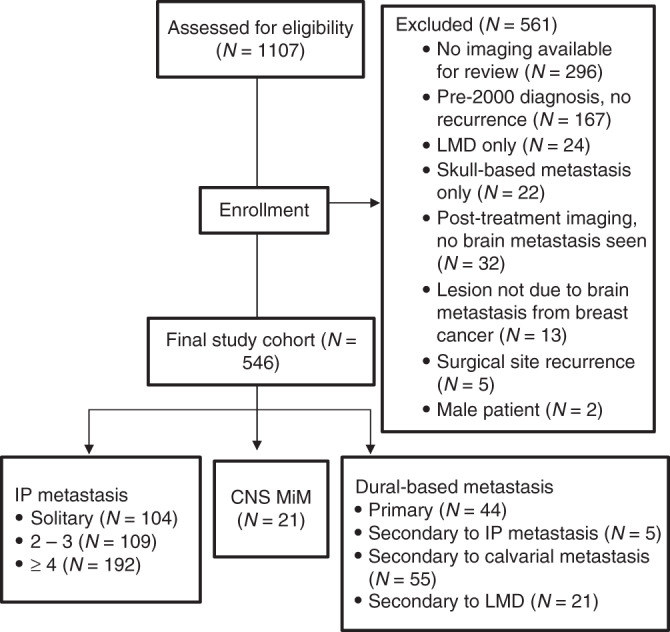
Flow chart of patients assessed for eligibility, those excluded, and final study cohort according to type of brain metastasis identified. IP intraparenchymal; CNS MiM central nervous system miliary metastasis; LMD leptomeningeal disease.

We assessed 1107 patients for eligibility; 561 were excluded, primarily due to inaccessibility of neuroimages for direct review. In all, 546 patients were included in our final study cohort, and these were stratified according to CNS metastatic subtype: traditional intraparenchymal metastases, dural-based metastases, and/or MiM (Fig. 1). The total number of CNS metastases recorded exceeded 546 because patients fell into more than 1 metastatic category.

Based on the proposed imaging criteria, 21 patients were identified with MiM (Table 1). Median age at the time of MiM development was 51 years, and the median time to MiM development was 35.5 months. In agreement with prior reports,^5–7,13–16^ our patient cohort also presented with largely nonspecific neurologic signs and symptoms at diagnosis, including headache, confusion, and/or gait disturbance. Only 1 patient (patient 15) was diagnosed with MiM incidentally, upon brain MR imaging that was ordered in follow-up for a prior skull-based metastasis and revealed innumerable punctate lesions meeting MiM radiographic criteria.

**Table 1:** Characteristics of patients with miliary metastasis, including breast cancer subtype, time to CNS and miliary metastasis development, symptoms at presentation, and treatment options pursued.

Patient	Age at MiM diagnosis, years	Breast cancer subtype	Time to CNS disease, months^a^	Time to MiM, Months^b^	CNS Sx	MiM Sx	Tx	Survival post MiM, months^c^
1	49	HR+/HER2+	44.2	44.2		HA	WBRT	10.8
2	63	TNBC	53.7	53.7		HA	Spine XRT	1.8
3	51	HR+/HER2+	70.4	71.5	HA	HA & Dizziness	WBRT	10.2
4	68	TNBC	35.5	35.5		Ataxia; LE Weakness	WBRT	0.5
5	63	HR+/HER2−	154.9	156	Ataxia	Increased Ataxia	WBRT	6.1*
6	40	HR+/HER2−	18.5	23.0	HA	LUE Numbness	WBRT	3.5
7	45	HR−/HER2+	26.3	29.3	HA	Aural Fullness	WBRT prior to MiM	3.8
8	42	HR−/HER2+	34.2	39.4	Seizure	Ataxia, HA, UE Weakness	WBRT	1.8
9	48	HR+/HER2+	22.2	33.2	HA, Ataxia	Ataxia	WBRT	13.3
10	46	TNBC	29.2	29.2		Diplopia, Rt Eye Deviation	Ocular XRT & WBRT	3.8
11	61	HR−/HER2+	3.8	3.8		N/V, Ataxia	WBRT	5.0
12	54	TNBC	132.5	132.5		Diplopia	WBRT	1.7
13	67	HR+/HER2−	89.6	89.6		RLE Numbness	WBRT	0.8*
14	32	TNBC	82.0	126.6	HA	Increased HA, N/V	Hospice	4.0
15	49	HR−/HER2+	25.0	25.0		Incidental due to skull lesion	WBRT	4.1
16	51	TNBC	22.2	22.4	LUE Weakness, Confusion	Seizure	WBRT	1.9
17	66	HR+/HER2−	131	131		Seizure	WBRT	9.8
18	71	TNBC	12.1	12.1		Confusion, Poor Appetite	Hospice	1.5
19	61	Unknown	63.5	65.5	HA, Dizziness	Confusion, Incontinence	WBRT prior to MiM	0.4
20	51	TNBC	24.5	24.9	Dizziness, Diplopia	HA	WBRT	3.7
21	31	HR−/HER2+	7.2	7.4	HA, Confusion	Seizure	WBRT	0.2
Median	51	N/A	34.2	35.5	N/A	N/A	N/A	3.7

*CNS* central nervous system, *HA* Headache, *HR+/HER2+* hormone receptor positive and human epithelial growth factor 2 positive, *HR+/HER2−* HR positive but HER2 negative, *HR−/HER2+* HR negative but HER2 positive, *LE* lower extremity, *LUE* left upper extremity, *MiM* miliary metastasis, *N/A* not applicable, *N/V* nausea and vomiting, *RLE* right lower extremity, *Rt* right, *Sx* symptom(s) at diagnosis, *TNBC* triple-negative breast cancer (HR−/HER2−), *Tx* therapy following MiM diagnosis, *UE* upper extremity, *WBRT* whole-brain radiation therapy, *XRT* radiation therapy.

^a^Time to CNS disease was calculated from time of breast cancer diagnosis to time of first CNS involvement.

^b^Time to MiM was calculated from time of breast cancer diagnosis to time of MiM diagnosis.

^c^Survival Post MiM was calculated from time of MiM diagnosis to time of death or date of last known follow up.

*Corresponds to patients who were lost to follow up. A date of death is not known.

**Fig. 2:**
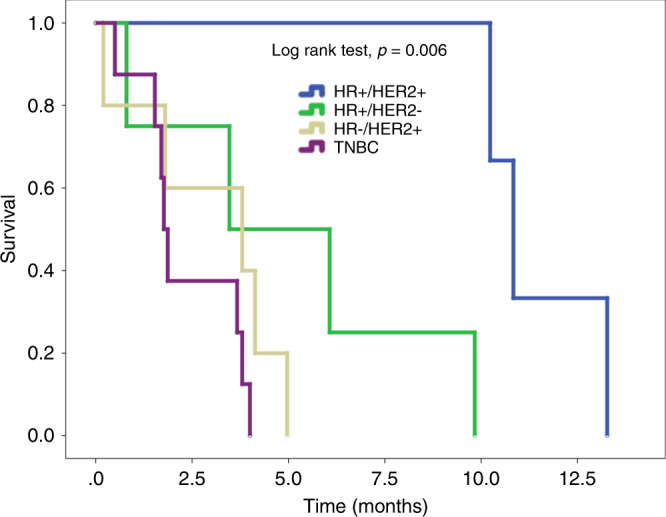
Kaplan–Meier curve representing survival after miliary metastasis diagnosis according to breast cancer subtype. HR+/HER2+ hormone receptor positive and human epithelial growth factor 2 positive; HR+/HER2− HR positive but HER2 negative; HR−/HER2+ HR negative but HER2 positive; TNBC triple-negative breast cancer (HR−/HER2−).

Eleven patients had a known prior history of CNS metastasis at the time of MiM diagnosis, while the remaining 10 were diagnosed with MiM as the initial CNS involvement; median time to MiM development after CNS metastasis was 1.3 months in these 11 patients. In all 11, repeat imaging was performed due to new neurological symptoms. Although the time between first known CNS diagnosis and development of MiM was as short as 0.2 months in 1 patient (patient 21), repeat brain MRI findings consistent with our definition of MiM were new (i.e., not seen in the prior MR imaging) in all 11 patients. Additionally, breast cancer subtype did not appear to have an impact on whether MiM developed following known CNS metastasis or as the first known CNS disease, as patients from all subtypes sorted into both categories. Median time to MiM development did not differ significantly across breast cancer subtypes (Table 2). HR+/HER2+ patients were found to have significantly longer overall survival following MiM diagnosis (10.8 versus 3.7 months; Table 2, Fig. 2).

**Table 2:** Disease outcomes according to breast cancer subtype.

Breast Cancer Subtype	Time to MiM, Median (Range), Months^a^ (*p* = 0.056)	Survival after MiM, Median (Range), Months^b^ (*p* = 0.006)
TNBC (*n* = 8)	32.3 (12.1–132.5)	1.8 (0.5–4.0)
HR+/HER2+ (*n* = 3)	44.2 (33.2–71.5)	10.8 (10.2–13.3)
HR+/HER2− (*n* = 4)	110.2 (23.0–156.0)	4.8 (0.8–9.8)
HR−/HER2+ (*n* = 5)	24.9 (3.8–39.4)	3.8 (0.2–5.0)
All^c^ (*n* = 21)	35.5 (3.8–156.0)	3.7 (0.2–13.3)

Log rank analysis was utilised to calculate statistical significance.

*HR+/HER2+* hormone receptor positive and human epithelial growth factor 2 positive, *HR+/HER2−* HR positive but HER2 negative, *HR−/HER2+* HR negative but HER2 positive, *TNBC* triple-negative breast cancer (HR−/HER2−), MiM miliary metastasis.

^a^Median time to MiM was calculated from time of breast cancer diagnosis to MiM diagnosis.

^b^Survival after MiM was calculated from time of MiM diagnosis to either known time of death or known last follow-up.

^c^One patient’s subtype was unknown.

Table 3 shows that most patients with MiM had documented metastatic disease prior to MiM development. Only four patients (patients 5, 6, 9 and 11) were diagnosed with CNS metastasis as a site of initial distant systemic metastasis, and only patient 9 had MiM at the time of diagnosis of initial distant metastasis; the remaining 17 patients all had distant systemic metastasis prior to CNS or MiM development. LMD, diagnosed by either cerebrospinal fluid analysis or imaging characteristics, was noted in 13 of the 21 patients. All 21 patients with MiM were treated per standard of care with whole-brain radiation therapy (WBRT) if feasible based on prior cumulative radiation doses and if they opted for WBRT rather than less aggressive measures. None of the patients underwent surgical resection of brain metastases and none of the patients with LMD and MiM received intrathecal chemotherapy; patient 2 received radiation to the spine for symptoms attributable to LMD rather than WBRT for MiM.

**Table 3:** Systemic therapies given to patients with miliary metastasis, along with time and site of first distant metastasis, and presence or absence of leptomeningeal disease.

Patient	Breast cancer subtype	Surgery	NACT (no. cycles)	Adjuvant CT (no. cycles)	Hormonal therapy	Radiation therapy	Time to 1^st^ Distant Metastasis, Months^a^	Site of 1^st^ Distant Metastasis	LMD^b^
1	HR+/HER2+	MRM	Docetaxel, Carboplatin, Trastuzumab (6)	Docetaxel Trastuzumab Carboplatin (2)	Adjuvant only; Anastrozole	Adjuvant only; Rt chest wall	39.7	Pleura	Y
2	TNBC	MRM		Doxorubicin, 5-FU, CP (6)	None	None	33.8	T LN	Y
3	HR+/HER2+	Mast w/ ALND		Doxorubicin, 5-FU, CP (6)	Adjuvant only; Tamoxifen	None	61.8	Bone, Lung, Liver	Y
4	TNBC	MRM			None	None	27.2	Lung, Bone	N
5	HR+/HER2-	MRM		Doxorubicin, 5-FU, CP (4)	Adjuvant only; Tamoxifen, Letrozole, Aromasin^c^	Adjuvant only; Lt breast	154.9	Liver, Brain	N
6	HR+/HER2−	Mast w/out ALND	Doxorubicin, CP, 5-FU, Paclitaxel (16)		Adjuvant only; Tamoxifen	Adjuvant only; Lt chest wall & peripheral lymphatics	18.5	Liver, Lung, Brain	Y
7	HR−/HER2+	MRM		Doxorubicin, 5-FU, CP (6)	None	None	17.9	Bone, T LN, Liver	Y
8	HR−/HER2+	MRM		CP, Doxorubicin (4)	None	None	29.0	Bone, Pleura, Lung	Y
9	HR+/HER2+	MRM			None	Adjuvant only; Lt chest wall & IMLN	22.2	Brain	N
10	TNBC	Seg Mast		Paclitaxel, CP, 5-FU, Doxorubicin (8)	None	Adjuvant only; Rt breast	27.4	Lung, T LN	Y
11	HR−/HER2+	None	Paclitaxel, CP, 5-FU, Epirubicin, Trastuzumab (10)		None	None	3.8	Brain, Meninges, SC, Bone	Y
12	TNBC	MRM		Doxorubicin, 5-FU, CP (8)	None	None	54.8	Liver, Ax LN	Y
13	HR+/HER2−	MRM			Adjuvant only; Tamoxifen, Raloxifene^d^	Adjuvant only; Lt breast	84.9	Pleura, Bone, T LN	N
14	TNBC	Wide re-excision post BCS		CP, 5-FU, MTX (6)	None	Adjuvant only; Rt breast	20.4	Lung	N
15	HR−/HER2+	MRM	Doxorubicin, CP, Paclitaxel, Carboplatin, Trastuzumab (10)		None	Adjuvant only; Rt chest wall	15.9	Bone	N
16	TNBC	Mast w/out ALND	Doxorubicin, CP, 5-FU, Paclitaxel (18)		None	Neoadjuvant only; Lt breast, Lt ALN & IFVLN	9.8	Lung	Y
17	HR+/HER2−	Lump w/ALND			Adjuvant only; Tamoxifen	Adjuvant only; Lt breast	105.0	Bone	Y
18	TNBC	Seg Mast			None	Adjuvant only; Lt breast	11.3	T LN, Liver, Lung, Pleura	N
19	Unknown	Seg Mast w/ALND		Doxorubicin, 5-FU, CP (6)	Adjuvant only; Tamoxifen	Adjuvant only; Lt breast	50.8	Liver, Lung	Y
20	TNBC	Mast w/out ALND			None	None	20.3	Liver, Lung, Bone	N
21	HR−/HER2+	None			None	None	0.9	Liver, BM	Y
Median	N/A	N/A	N/A	N/A	N/A	N/A	27.2	N/A	N/A

*5-FU* fluorouracil, *Adjuvant CT* adjuvant chemotherapy regimen, *Ax LN* axial lymph node, *BM* bone marrow, *CP* cyclophosphamide, *HR+/HER2+* hormone receptor positive and human epithelial growth factor 2 positive, *HR+/HER2− HR* positive but HER2 negative, *HR−/HER2+* HR negative but HER2 positive, *LMD* leptomeningeal disease, *MiM* miliary metastasis, *MTX* methotrexate, *NACT* neoadjuvant chemotherapy regimen, *SC* spinal cord, *TLN* intrathoracic lymph node, *TNBC* triple-negative breast cancer (HR−/HER2−), *MRM* modified radical mastectomy, *Mast w/ALND* mastectomy with axillary lymph node dissection, *Mast w/out ALND* mastectomy without axillary lymph node dissection, *Seg Mast* segmental mastectomy, *BCS* breast conserving surgery, *Lump w/ALND* lumpectomy with axillary lymph node dissection, *Seg Mast w/ALND* segmental mastectomy with axillary lymph node dissection, *Rt* right, *Lt* left, *IMLN* inframammary lymph node, *ALN* axillary lymph node, *IFVLN* infraclavicular lymph node.

^a^Time to first distant metastasis refers to time from breast cancer diagnosis to development of first distant metastasis.

^b^LMD presence was determined by imaging or cerebrospinal fluid analysis.

^c^Patient received Tamoxifen as initial hormonal therapy followed by Letrozole as second hormonal therapy, and then Aromasin as final hormonal therapy regimen.

^d^Patient received Tamoxifen as initial hormonal therapy followed by Raloxifene as final hormonal therapy regimen.

## Discussion

Largely reported in sporadic autopsy analyses of patients who present with various neurologic symptoms and imaging findings of multiple, small brain metastases,^5,6^ MiM has thus far been a poorly understood subset of brain metastatic disease. When Madow and Alpers^4^ first reported their findings in autopsy analyses of patients with solid malignancies of different origins, they estimated the incidence of MiM at 3.8%, having noted this entity in 4 out of 106 patients evaluated. However, following their report in 1951, limited follow-up studies have been conducted to further elucidate disease-specific clinicopathologic parameters or outcome measures.

Arguably, the study of MiM has been limited by the lack of antemortem, objective, diagnostic radiographic criteria, which has likely contributed to a lack of awareness and underreporting of this metastatic subtype, and to the lack of a standardised definition in studies that have evaluated MiM. Although MiM has thus far been reported overwhelmingly in lung and gastrointestinal primary cancers, the present analysis establishes the presence of MiM in breast cancer, with an incidence in our review of 3.8%, and the present study is the first retrospective analysis to develop objective radiographic criteria for the diagnosis of MiM, as well as providing clinicopathologic correlates drawn from a standardised cohort of MiM patients. Interestingly, it is noted that the incidence in our review matched exactly that postulated by Madow and Alpers^4^ in 1951. It is unclear if this proportion signifies a true incidence of MiM across various cancer subtypes, as none of the four patients evaluated by Madow and Alpers^4^ were known to have breast cancer (three patients had bronchogenic carcinoma of the lung and one patient had carcinoma of an unknown primary site), or if the incidence in our report matches that of Madow and Alpers^4^ by coincidence. As such, additional studies with larger patient populations are needed to investigate and elucidate these clinical queries and data.

Upon our initial review of breast cancer patients with brain metastasis, patterns emerged from radiographic image analysis that raised questions concerning the possibility of multiple aetiologies and/or subtypes of brain metastases in the cohort. Five hundred forty-six patients with brain metastasis from primary breast cancer were included in the study following individual review of neuroradiographic imaging studies. As seen in Fig. 1, the majority of patients initially evaluated were determined to have traditional intraparenchymal lesions, identified as those spherical in shape and commonly distributed at the grey-white junction, with or without surrounding vasogenic oedema.^3^ Surprisingly, although MiM has not been widely reported in breast cancer, our review of the neuroradiographic images identified a small but significant subset of patients whose brain metastases matched the descriptions of MiM in prior reports of other systemic malignancies.^17^

Due to the fact that imaging characteristics were not uniform, and given the lack of a standardised radiographic definition for MiM, identifying patients with MiM, as opposed to those with numerous conventional intraparenchymal lesions, proved challenging. Unlike prior reports based on post-mortem autopsy analyses of patients thought to have MiM,^5,6,12^ our patients did not have autopsies or histopathologic CNS specimens for evaluation, necessitating our development of radiographic criteria for the identification of MiM. The finding of numerous, punctate lesions in a perivascular distribution, and with little mass effect in several patients, heightened our suspicion for MiM in a number of cases, and review of the imaging by a neuroradiologist discerned the patterns and characteristics that differentiated cases of MiM from those believed to be simply numerous traditional intraparenchymal metastases.

**Fig. 3:**
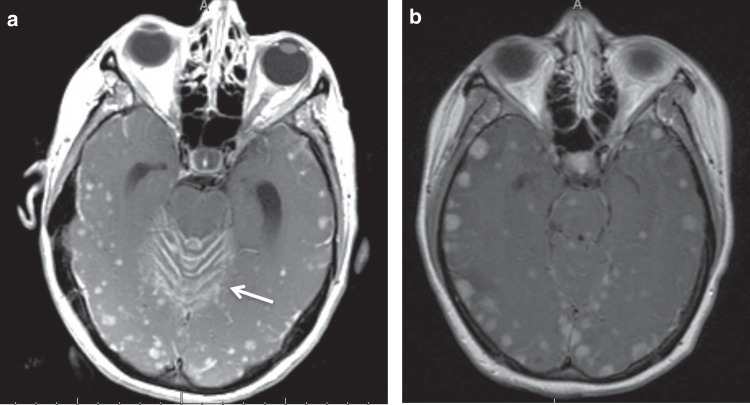
Patients with typical miliary metastatic lesions seen on neuroimaging. **a** Example of miliary metastasis with concomitant imaging diagnosis of leptomeningeal disease (LMD denoted by arrow). **b** Example of miliary metastasis with lesions of various sizes, reinforcing our decision to include lesion number and anatomical distribution in our radiographic definition but not rely on lesion size.

Often, patients in our review had both CT and MR brain imaging studies performed, and upon the review of these, radiographic criteria for the identification of MiM on both CT and MR images were postulated. In agreement with prior descriptions of MiM,^5,10,17^ the anatomical distribution of nodules proved to be a defining characteristic. Not only did lesions have to be located bilaterally, but they had to be located bilaterally in both the infratentorial and supratentorial compartments as well, hence a “4 quadrant” disease. Lesion number also contributed to the radiographic definition of MiM. MiM has been previously quantified simply as “multiple” or “innumerable” in case reports; we discerned that MiM was characterised on MR imaging by the presence of 20 or more lesions per imaging slice on at least 2 non-contiguous slices, and on CT by 10 or more lesions per slice on at least 2 non-contiguous slices (accounting for the reduced spatial resolution and the resulting decreased conspicuity of CT imaging in identifying smaller CNS lesions). Of note, lesion size was determined to not be a reliable criterion for identification of MiM, as patients meeting the anatomical and numerical criteria for MiM could have lesions of varying size (Fig. 3). Two of the 21 MiM cases were diagnosed on CT imaging alone, without MR imaging available for comparison. One patient (patient 11), who presented with acute gait instability, underwent initial diagnostic work up with CT imaging and follow-up MR imaging for confirmation. In this patient, CT findings met our radiographic criteria and were again confirmed on MRI. Although our analysis is limited by the lack of histopathologic examination for validating our proposed radiographic criteria, these numerical and anatomical criteria ultimately allowed for a practical and reasonable method to discern between cases of MiM and those thought to be numerous, but non-miliary, metastasis. Utilising our radiographic criteria as an initial guide to potentially standardise MiM evaluation, future studies with larger datasets, histopathologic confirmation, and consensus image interpretation are all needed to validate our diagnostic criteria.

Our study is the first to establish the incidence of MiM in breast cancer at roughly 3.8%. The pathogenesis and mechanism of MiM metastasis is poorly understood. With the identification of 21 MiM patients, we noted that 11 had known CNS metastasis at the time of MiM diagnosis, while the remaining 10 were diagnosed with MiM as the initial CNS involvement from their primary breast cancer (Table 1). To the best of our knowledge, no prior report has documented the development of MiM in patients with a history of CNS metastasis. This series enables a unique longitudinal radiographic evaluation of MiM, allows for the comparison of imaging before and after the development of MiM, and offers possible insights into MiM dissemination. The time between first known CNS diagnosis and development of MiM was as short as 0.2 months in one patient, and findings on repeat neuroimaging consistent with the proposed defining criteria for MiM were not present on prior neuroimages in all 11 patients, raising the question as to whether metastasis in MiM develops as an acute showering event. Although the lack of histopathological examination in this analysis limits our direct evaluation of MiM lesions and hinders our ability to further postulate mechanisms of metastasis, prior analyses have offered various hypotheses to explain the unique distribution of MiM in the CNS.

Bugalho et al.^12^ reported the first case of MiM from primary small cell gastric carcinoma. Although previously unknown in small cell gastric cancer, MiM had been reported in patients with small cell lung cancer. Therefore, Bugalho et al.^12^ postulated the seed-and-soil hypothesis and suggested that similar cell types may have an underlying similarity in biological behaviour and predilection for metastatic patterns. Furthermore, in 117 metastatic foci from 14 samples, Ogawa et al.^6^ noted that carcinoma cells were at least initially located exclusively in the perivascular (Virchow-Robin)/subpial space compartment, not in the brain parenchyma proper or in the subarachnoid space. Given this observation, Ogawa et al.^6^ postulated a sequence of metastatic progression in which tiny foci of metastasis initially gained access to the perivascular space compartment of the middle cerebral neocortical grey ribbon layer (lamina 3) and then proceeded to spread centrifugally within that compartment to eventually reach the contiguous subpial space superficially and the subcortical white matter subjacently. The pathobiology of this intriguing process remains to be elucidated.

Despite the limited understanding of the mechanisms of metastasis, our analysis is the first to offer clinical correlates and outcome measures in MiM. In this regard, MiM was noted in patients with all breast cancer subtypes, and, consistent with prior reports of MiM in other systemic malignancies,^5–7,13–16^ patients presented with nonspecific neurologic signs and symptoms. Breast cancer subtype did not affect whether MiM developed following known CNS metastasis or as the first known CNS disease, and the median time to MiM development did not differ significantly across breast cancer subtypes. Patients with HR+/HER2+ disease were found to have significantly longer survival following MiM diagnosis. Given the limited sample size when comparing breast cancer subtypes, these findings need to be further evaluated in future studies with larger patient populations.

As only 4 of 21 patients in our review were diagnosed with CNS metastasis as a site of initial distant metastasis, and only 1 of those patients had MiM at the time of diagnosis of initial distant metastasis, it can be inferred that MiM may be a manifestation of progressive disease, and it appears to be a late site of metastasis in breast cancer (Table 3). This finding may largely be due to the administration of systemic treatments and additional therapies, such as surgery, neoadjuvant and/or adjuvant chemotherapy, as well as hormonal therapy and radiation where indicated (Table 3). Furthermore, with the median time to MiM development approaching 3 years, but with a median survival after MiM at only 3.7 months regardless of therapy offered, questions arise regarding how to best clinically approach MiM.

All patients in our review received WBRT if feasible on the basis of prior cumulative radiation doses unless they opted for less aggressive care. LMD was noted in several patients, either on imaging or through the presence of malignant cells in the cerebrospinal fluid, but treatment was limited to WBRT. Because LMD, much like MiM, is commonly associated with vague neurological symptoms, such as headache, cranial nerve and/or focal neurological deficits and seizure,^18–20^ and also imparts a grave prognosis that is measured in weeks-to-months,^21^ MiM may be clinically more similar to LMD than to intraparenchymal brain metastasis. Given these observed similarities between MiM and LMD, future studies should evaluate whether the treatment of MiM is best approached as an LMD-like condition by pursuing radiation therapy, intrathecal chemotherapy, and/or systemic chemotherapy, rather than limiting treatment to options that target innumerable brain metastases.

At this time, it is unknown the degree to which, if at all, MiM represents a distinct clinical entity from intraparenchymal brain metastasis or rather a manifestation of a more aggressive disease process. The pathophysiology behind MiM spread, although postulated to be different from traditional intraparenchymal metastasis, is yet to be fully elucidated, and our observation of clinical similarities to LMD do raise several unanswered questions. Sample size, along with the lack of confirmatory histopathological examination, are the largest limitations in the present analysis, and the scope of future research should be directed to address these numerous knowledge gaps in an effort to better understand MiM as a clinical entity. Nevertheless, the present review is the first to identify clinical and imaging characteristics in MiM that may help guide future CNS metastasis research.

## Data Availability

No publicly available data was utilised in this study. All data utilised is from the prospectively maintained patient database in the Department of Breast Medical Oncology at the University of Texas MD Anderson Cancer Center. All data was handled in accordance with regulations put forth by the Health Insurance Portability and Accountability Act to ensure that no protected health information for participants in this study was inappropriately handled or disclosed.
